# Editorial: Refractory Pituitary Adenoma—Current Challenges and Emerging Treatments

**DOI:** 10.3389/fendo.2022.868174

**Published:** 2022-03-09

**Authors:** Renzhi Wang, Cuiqi Zhou, Ann I. McCormack, Adam N. Mamelak

**Affiliations:** ^1^ Department of Neurosurgery, Peking Union Medical College Hospital (CAMS), Beijing, China; ^2^ Department of Medicine, Cedars-Sinai Medical Center, Los Angeles, CA, United States; ^3^ Department of Endocrinology, St Vincent’s Hospital Sydney, Darlinghurst, NSW, Australia; ^4^ Department of Neurosurgery, Cedars-Sinai Medical Center, Los Angeles, CA, United States

**Keywords:** refractory pituitary adenomas, diagnosis, surgical treatment, medical therapy, temozolomide, targeted and immunotherapy, drug resistance, pathogenesis

Refractory pituitary adenomas (PAs) are defined as aggressive-invasive PAs characterized by rapid growth, frequent recurrence, a high Ki-67 index, and resistance to standard therapeutic approaches (1, Dai et al.). It is notoriously difficult to manage these refractory PAs due to limited efficacy of current therapeutic options. After initial treatment, refractory PAs frequently re-grow or recur and require continued therapies including medical therapy, radiotherapy, and re-operation. However, these patients with refractory PAs often do not respond to these treatments and have a very poor prognosis (2). Recently, temozolomide (TMZ) has shown moderate efficacy for refractory PAs and pituitary carcinomas (PCs) (3). In 2018, the European Society of Endocrinology Clinical Practice Guidelines recommended TMZ as the first-line treatment for refractory PAs and PCs when conventional treatments have failed to control tumor growth (4). However, many refractory PAs demonstrate only transient response to TMZ therapy, and subset of them develop drug resistance during treatment (5, 6). Although targeted therapies such as anti-epidermal growth factor receptor (EGFR), anti-vascular endothelial growth factor (VEGF), and inhibitors of mammalian target of rapamycin (mTOR) signal pathway have also been used to treat refractory PAs and PCs, effectiveness of these targeted therapies is still largely unknown due to a lack of data from preclinical research and clinical trials (7, 8). Cancer immunotherapy is also a promising treatment option for refractory PAs and PCs, but more basic research and clinical trials are needed to further verify its efficacy (Dai et al., 9).

In the present Research Topic in Frontiers in Endocrinology, we collected 16 studies and reviews on recent advancements of preclinical research and clinical cases in diagnosis and treatment of refractory PAs.

Eight articles of clinical findings and case reports addressed imaging, immunotherapy, fluconazole and TMZ treatment of refractory pituitary tumors, and raised attention on silent corticotroph adenoma (SCA). Yan et al. showed that increased distance between the bilateral internal carotid arteries (ICAs) is related to disease duration, however, is not associated with preoperative levels of GH and IGF-1 in acromegalic patients. Both longer disease duration and refractory clinical presentation are risk factors of increased ICAs distance in acromegalic patients. Furthermore, Zheng’s and Zhang’s groups addressed SCA, a high-risk aggressive subtype of refractory PA in 2 separate articles. Zheng et al. retrospectively investigated the characteristics of SCA that transformed to typical Cushing’s syndrome at a single medical center and reviewed relevant literatures. All cases that converted from SCA to adrenocorticotropic hormone (ACTH)-secreting tumors were macroadenomas and the median time of conversion was 30 (13.0, 68.3) months. Once transformed, the ACTH-secreting tumors exhibited higher recurrence frequency, increased invasiveness and more serious hypercortisolism. Zhang et al. explored differences of clinical features and surgical outcomes between ACTH-negative and ACTH-positive SCA patients with positive Tpit immunostaining but no evidence for Cushing’s Syndrome. These findings indicated that the prevalence of SCA is substantially underestimated, thus more attention and long-term follow-up is important for SCAs. Moreover, Lamb et al. reported a case of PC responding to immune checkpoint inhibitor (ICI) therapy and subsequent anti-VEGF therapy. The PC presented satisfied initial response to ICI therapy combining anti-PD-1 and anti-CTLA4. Survival was lengthened and morbidity reduced in the patient. This case raised the potential of sequential or combining ICI and anti-VEGF therapy as a possible strategy treating aggressive pituitary tumors. Xiang et al. described a case of autoimmune hypophysitis (AH) coexisting with systemic lupus erythematosus (SLE) and summarized all case reports of AH with SLE. After treatments with immunosuppressant and glucocorticoids, symptoms of hyponatraemia and hypopituitarism gradually disappeared, and long-term remission was achieved. This case report implied a potential relationship between SLE and AH, and AH should be concerned when evaluating patients with SLE. Zhao et al. reported a case of a patient with Cushing’s disease and pulmonary cryptococcus neoformans treating with fluconazole, both cryptococcus infection and hypercortisolism were relieved. Therefore, as a possible alternative treatment, fluconazole may be effective for patients with Cushing’s disease and cryptococcal pneumonia. Tang et al. presented a case of aggressive prolactinoma that was resistant to dopamine agonists treatment but responded to TMZ. After a total of six-cycle TMZ treatment, tumor was completely disappeared and PRL level normalized. Authors further performed genome sequencing to investigate the underlying mechanism of the tumor aggressiveness and high sensitivity to TMZ. Additionally, Cooper et al. summarized 9 cases of aggressive PAs to demonstrate the importance and significance of multidisciplinary, individualized treatment. The choice of treatment timing and rationality for different therapeutic approaches in each case were reviewed, as well as strategies of individualized treatment were discussed.

Three preclinical and basic studies investigated mechanisms underlying refractory/aggressive PAs. Wang et al. studied the mechanism underlying miR-134 effect on alleviating growth of nonfunctioning pituitary adenoma cells. miR-134 significantly inhibited αT3-1 cell proliferation, VEGFA expression, and G1/S cell cycle transition. This inhibition effect can be reversed by stromal cell-derived factor-1alpha and overexpressed VEGFA. In primary nonfunctioning PAs, miR-134 expression level negatively correlated with invasiveness of tumor. These findings implied a novel mechanism of pituitary tumor pathogenesis and provided potential therapeutic target. Moreover, Shen et al. discovered the role of heat-shock protein 90 (Hsp90) in ACTH-secreting adenomas cells and the possible clinical application of Hsp90 inhibitor 17-N-allylamino-17-demethoxygeldanamycin (17-AAG) in Cushing’s disease. 17-AAG attenuated cell viability and decreased secretory function of human ACTH-secreting adenomas cells. Inhibitory effect of 17-AAG on USP8 mutant tumor cells were increased than the effect on USP8 wild-type cells, indicating 17-AAG as a potential treatment option for Cushing’s disease. Furthermore, Xi et al. explored significant differences of immunologic profiles in normal pituitaries vs PAs, and in benign vs aggressive PAs. Authors quantitatively analyzed mRNA expression levels of CD86, CD80, CLTA-4, PD-1, PD-L1 and PD-L2. Aggressive PAs showed increased CD80, CD86 and PD-L2 than normal pituitaries, as well as elevated CD80 and CD86 than non-aggressive PAs. These results suggested scientific rationale of ICI therapy for PAs.

Several review articles discussed current progress of refractory PA treatment and addressed new hypotheses for PC. Dai et al. addressed recent progress and current controversies of the classification and definition of pituitary tumors. Terms and classifications of PitNET, aggressive PAs and refractory PAs were discussed. Authors suggested that the term of refractory PAs accurately represents characteristics of these tumors, and the diagnostic criteria are more strict, objective, and accurate. This diagnostic criterion may help to early identify patients with refractory PAs and adopt aggressive therapeutic strategies, therefore achieve better clinical outcomes. Dai et al. also summarized the progress of immunotherapy in refractory PAs and PCs. The expression of immune factors including PD-1, PD-L1 macrophages, and lymphocytes were significantly related to clinicopathological characteristics of PAs. Clinical studies suggested the benefit of immunotherapy for refractory PA or PC patients, while more preclinical study and clinical trials are required to further elucidate the efficacy. Furthermore, Nakano-Tateno et al. provided comprehensive review on the efficacies and protocols of medical treatments for aggressive PAs. Aggressive PAs can be resistant to conventional therapies. Some novel therapeutic strategies, such as TMZ, TMZ-Based combination therapies (CAPTEM), inhibitors of mTOR signal pathway and CDK2, anti-EGFR, immunotherapy, peptide receptor radionuclide therapy (PRRT), and retinoic acid were discussed in this review. In addition, Yamamoto et al. introduced several aggressive types of ACTHomas including Crooke’s cell adenoma (CCA), Nelson’s syndrome and SCAs, summarized current knowledge of the definition, pathophysiology, and treatment of refractory ACTHomas, and provided directions for future research. Moreover, Dai et al. proposed the hypothesis of metastatic spread of PCs. Authors reviewed previously reported cases of PCs and surmised that different secretory types of PCs prefer their own favorable metastatic organs through different patterns of metastatic spread. Therefore, the “seed and soil” theory could be adopted to explain the metastatic pattern of PCs.

In summary, studies on diagnosis and treatment of refractory PAs have achieved significant advances. However, this Research Topic only contains a limited fraction of many important aspects of refractory PAs. We expect future issues will collect more studies on basic and translational research and large-scale clinical trials, bringing a better understanding of characteristics and underlying mechanisms of refractory PAs. Moreover, new therapeutic concepts and strategies such as personalized and precision medicine may also play a role in future treatment. However, a significantly more preclinical and clinical research is needed to further understand the nature of the mechanism underlying refractory PA growth, and to improve the clinical outcomes for patients suffering with these tumors.

## Author Contributions

RW and CZ drafted this manuscript. AIM and ANM polished and revised the text. All authors contributed to the article and approved the submitted version.

## Conflict of Interest

The authors declare that the research was conducted in the absence of any commercial or financial relationships that could be construed as a potential conflict of interest.

The handling editor declared a past collaboration with several of the authors (AIM, ANM).

## Publisher’s Note

All claims expressed in this article are solely those of the authors and do not necessarily represent those of their affiliated organizations, or those of the publisher, the editors and the reviewers. Any product that may be evaluated in this article, or claim that may be made by its manufacturer, is not guaranteed or endorsed by the publisher.
